# Supplementary figures and images for: Indirect effects of overfishing on Caribbean reefs: sponges overgrow reef-building corals
Source: PeerJ. 2015 Apr 28;3:e901. doi: 10.7717/peerj.901 (PMC4419544; doi:10.7717/peerj.901)

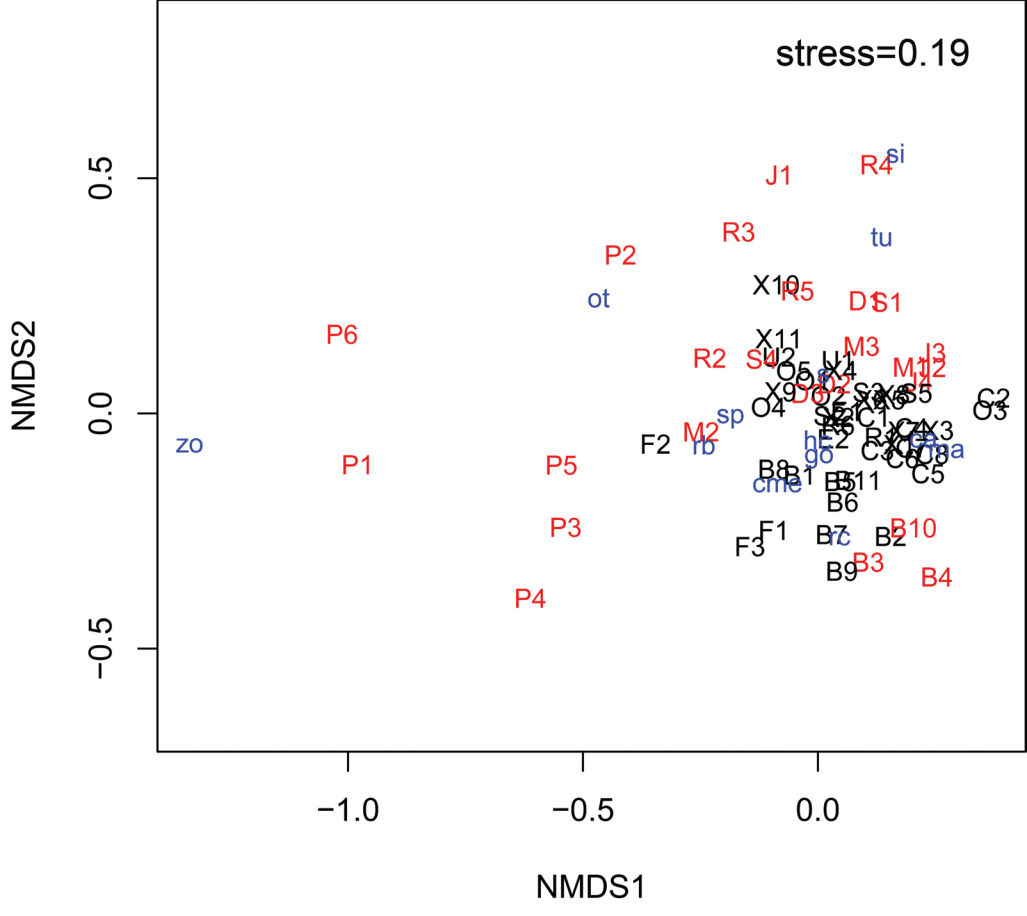

Supplement: Figure S1 — Occurrence is the number of times each category appears per 625 points of each transect. Sites labeled black are less-fished, and sites labeled red are overfished. Factors labeled in blue. The following benthic categories were used: ca, coralline algae; cme, fire coral; go, gorgonian; hc, hard coral; ma, macroalgae; ot, other benthos; rc, rock; rb, rubble; s, sand; si, silt; sp, sponges; tu, turf; and zo, zoanthid. Prefixes of site names denote the following locations: B, Bahamas; C, Cayman Islands; D, Dominican Republic; E, St. Eustatius; F, Key Largo, FL; J, Jamaica; M, Martinique; O, Bonaire; P, Bocas del Toro, Panama; R, Puerto Rico; S, St. Lucia; U, Curaçao; X, Mexico. [file peerj-03-901-s001.pdf]
