# Supplementary material for: Indirect effects of overfishing on Caribbean reefs: sponges overgrow reef-building corals
Source: PeerJ. 2015 Apr 28;3:e901. doi: 10.7717/peerj.901 (PMC4419544; doi:10.7717/peerj.901)
Supplement: Table S2 [file peerj-03-901-s003.docx]

| **Benthic categories** | **Axis 1** | **Axis 2** |
| --- | --- | --- |
| Turf | 0.08 | **0.86** |
| Coralline algae | 0.50 | -0.12 |
| Fire coral (*Millepora* spp.) | -0.14 | -0.05 |
| Rock | -0.09 | **-0.64** |
| Gorgonian | -0.13 | 0.06 |
| Hard coral | -0.16 | 0.08 |
| Macroalgae | **0.80** | -0.30 |
| Other benthos | -0.40 | 0.22 |
| Rubble | -0.55 | -0.21 |
| Sand | -0.17 | 0.24 |
| Silt | 0.00 | 0.47 |
| Zoanthid | **-0.74** | -0.04 |
| Sponge | **-0.86** | 0.02 |
